# Bridging Disciplines: Applications of Forensic Science and Industrial Hemp

**DOI:** 10.3389/fmicb.2022.760374

**Published:** 2022-04-11

**Authors:** Sheree J. Finley, Gulnaz T. Javan, Robert L. Green

**Affiliations:** Department of Physical Sciences and Forensic Science Programs, Alabama State University, Montgomery, AL, United States

**Keywords:** industrial hemp (*Cannabis sativa* L.), hemp microbiome, microbial diversity, tetrahydrocannabinol, cannabidiol, forensic analyses

## Abstract

Forensic laboratories are required to have analytical tools to confidently differentiate illegal substances such as marijuana from legal products (i.e., industrial hemp). The Achilles heel of industrial hemp is its association with marijuana. Industrial hemp from the *Cannabis sativa* L. plant is reported to be one of the strongest natural multipurpose fibers on earth. The *Cannabis* plant is a vigorous annual crop broadly separated into two classes: industrial hemp and marijuana. Up until the eighteenth century, hemp was one of the major fibers in the United States. The decline of its cultivation and applications is largely due to burgeoning manufacture of synthetic fibers. Traditional composite materials such as concrete, fiberglass insulation, and lumber are environmentally unfavorable. Industrial hemp exhibits environmental sustainability, low maintenance, and high local and national economic impacts. The 2018 Farm Bill made way for the legalization of hemp by categorizing it as an ordinary agricultural commodity. Unlike marijuana, hemp contains less than 0.3% of the cannabinoid, Δ^9^-tetrahydrocannabinol, the psychoactive compound which gives users psychotropic effects and confers illegality in some locations. On the other hand, industrial hemp contains cannabidiol found in the resinous flower of *Cannabis* and is purported to have multiple advantageous uses. There is a paucity of investigations of the identity, microbial diversity, and biochemical characterizations of industrial hemp. This review provides background on important topics regarding hemp and the quantification of total tetrahydrocannabinol in hemp products. It will also serve as an overview of emergent microbiological studies regarding hemp inflorescences. Further, we examine challenges in using forensic analytical methodologies tasked to distinguish legal fiber-type material from illegal drug-types.

## Introduction

Forensic science laboratories work police departments to help authorities distinguish between industrial hemp and marijuana plants, which appear identical but confer very different legal statuses. Industrial hemp, a variety of *Cannabis sativa* L., is reported to be one of the stiffest and strongest natural fibers on earth (Pickering et al., 2007; Salentijn et al., 2019). It is also purported to be one of the earliest plants cultivated by man for medicinal purposes (Zuardi, 2006). *Cannabis* from the Cannabaceae family is the genus name for plants that are broadly separated into two classes: industrial hemp and marijuana. These multifunctional, herbaceous products have been farmed by mankind for millennia. Up until the eighteenth century, hemp was one of the major fibers in the United States. *Cannabis* spp. are the only plants that produce a unique class of molecules known as cannabinoids, specifically Δ^9^-tetrahydrocannabinol (THC) and cannabidiol (CBD; Hillig and Mahlberg, 2004; Zuardi, 2006; Battistella et al., 2014; Shover and Humphreys, 2019; Quaicoe et al., 2020; Schumacher et al., 2020; Adhikary et al., 2021). The Agricultural Improvement Act of 2018 (the 2018 Farm Bill) effectively exempted hemp from the list of federal Schedule I substances under the Controlled Substances Act (Shover and Humphreys, 2019). The incipient bill allowed for universities to research industrial hemp and for companies to commercially grow it in states with regulatory oversight that permits it to be produced. It also made way to produce hemp derivatives that contain less than 0.3% THC, the psychotropic cannabinoid found in marijuana. However, any hemp derivative that exceeds the 0.3% threshold is defined as marijuana and confers illegality as a Schedule I narcotic. The potential illegality of hemp material makes industrial hemp of particular importance in the field of forensic science.

Currently, industrial hemp is grown legally in more than 30 countries as a sustainable, eco-friendly, and multifunctional plant. It has high nutrient and water use efficiency and a superior biomass quality for textile and construction resources (Crini et al., 2020). Traditional building materials such as concrete, fiberglass insulation, and lumber pose valid considerations regarding their environmental impact and toxicity; however, industrial hemp exhibits ecological sustainability, low maintenance, and high impact for local and national economies. For example, hemp fiber production presents a low ecological footprint of 1.46–2.01 global hectares (gha; Schumacher et al., 2020). Industrial hemp has recently garnered an increased interest in the United States, and in 2022 it is expected to attain an annual revenue growth rate of 18.4% (Quaicoe et al., 2020). Currently, as this review will show, the topics of hemp-associated microbiome and fungi intended for beneficial cultivation are emerging as one of great interests.

This work aims to provide the findings in research focusing on the industrial hemp microbiome and the implications of these finding on hemp applications. This review also covers emerging topics of the pharmaceutical capabilities of hemp especially in light of the recent severe acute respiratory syndrome (SARS)-coronavirus-2 (COVID-19) pandemic. Further, an overview of the scientific literature is presented on forensic techniques involving differentiating legal hemp from illegal marijuana and the challenges that accompany such determinations.

## Differentiation Between Industrial Hemp and Marijuana

**Figure 1:**
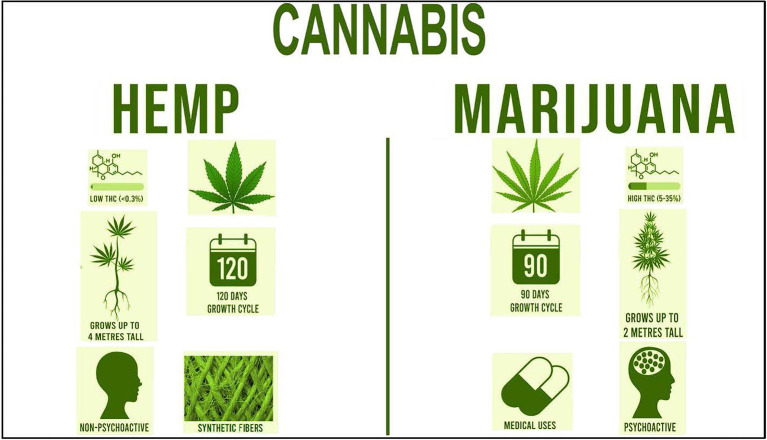
Comparison of the two varieties of Cannabis: industrial hemp and marijuana. The general leaf structure, percent levels of THC, psychoactive properties, growth height, growth cycle days, and uses are shown.

The 2018 Farm Bill, signed into law on 20 December 2018, made way for the legalization of industrial hemp by categorizing it as an ordinary agricultural commodity (Hillig and Mahlberg, 2004; Zuardi, 2006; Battistella et al., 2014; Shover and Humphreys, 2019; Quaicoe et al., 2020; Schumacher et al., 2020; Adhikary et al., 2021). Industrial hemp along with marijuana are two varieties of *Cannabis sativa* L. The primary distinction between industrial hemp and marijuana is the threshold concentration of the cannabinoid, THC, the psychoactive component that gives users psychotropic effects (Shover and Humphreys, 2019; Figure 1). Unlike marijuana, hemp contains less than 0.3% based on dry weight of THC in leaves and buds. According to the 2018 Farm Bill, if THC levels exceed this threshold, it is then classified as marijuana, the illegal plants are destroyed, and the grower faces the possibility of prosecution (Shover and Humphreys, 2019). Both plants are similar in appearance; however, hemp plants, with a 120-day growth cycle, tend to grow taller and have thinner leaves than marijuana plants and are harvested approximately 5 weeks prior to marijuana (Sankari and Mela, 1998). As an industrial product, marijuana fibers contain low tensile strength that break and shred easily; thus, it is not suitable for legal industrial applications.

Although, legally grown industrial hemp contains low levels of THC, it contains high levels of the CBD, the non-intoxicating compound extracted from the resinous flower of *Cannabis*. It contains many volatile compounds including terpenes which bestows a distinctive odor, terpenoid-like compounds, and cannabinoids which are extracted through steam distillation (Turner et al., 1980). These compounds interact directly with cannabinoid receptors located in the endocannabinoid system (Di Marzo, 2008), which bind human endogenous cannabinoids as well as exogenous plant-derived and synthetic cannabinoids. These molecules help modulate chronic inflammatory conditions and regulate immune homeostasis. CBD is purported to have multiple advantageous therapeutic and medicinal uses and does not demonstrate the psychotropic and euphoric effects exhibited by THC.

## Hemp Growth

Industrial hemp is very sensitive to ecological conditions, including photoperiod (length of day), plant density, harvest time, irrigation, soil nutrients, and temperature; therefore, cultivar genotypes are typically produced in specific environments for certain hemp applications. The plant is a branching herb and has a rigid, herbaceous stalk with varieties that come in an assortment of heights: 3–4 ft. (0.91–1.2 m; dwarf tall), 4–5 ft. (1.2–1.5 m; semi-dwarf tall), and 6–7 ft. (1.8–2.1 m; medium height; Small, 2015). The plant contains nodes at intervals of 10 to 50 cm. The leaflets are dark green, serrated, and taper at each end in 5 to 11 points (Small, 2015). The leaves grow up to 15 cm in length and 12 cm in width (Ehrensing, 1998; Kraenzel et al., 1998). The spindle-shaped central root grows to depths of 2–2.5 m and branches up to 80 cm in width.

Prior to the formation of flowers, hemp plants are dioecious, having both female and male reproductive organs and are visually indistinguishable (Onofri and Mandolino, 2017). The diploid plant includes both dioecious genotypes containing heteromorphic sex chromosomes and monoecious types with homomorphic sex chromosomes. It is primarily open-pollinated, that is, it is wind-pollenated. Female (pistillate) hemp plants are dense and produce raceme which looks similar to a candle-like clusters of flowers, whereas male (staminate) plants are tall and slender with fewer leaves surrounding the terminal, branched inflorescence. Sexual expressions are determined by abiotic and biotic environmental stressors (Onofri and Mandolino, 2017). For example, dry soils, extreme temperatures, low soil nitrogen concentrations, and low light intensities decrease the female: male plant ratio (Freeman et al., 1980). Male plants flower and senesce earlier than females (Struik et al., 2000). After flowers form, male hemp plants grow slenderer with elongated internodes at their tops. Male plants are more advantageous for industrial uses due to their production of a finer fiber (Salentijn et al., 2019). Although CBD is present in both male and female plants, most are found in resin glands on trichomes of the female flower buds (Mahlberg and Kim, 2004). The terpenophenolic compounds are secreted from head cells of trichome glands, specifically from the capitate-stalked glandular hairs (Happyana et al., 2013). Female flowers require mild temperatures and high nitrogen concentrations and light intensity. They are often propagated in greenhouses from feminized seeds or female clones and then transplanted two to 4 weeks after establishing growth (Adesina et al., 2020).

Hemp grows optimally in mild, humid climates; however, the ideal growing temperatures ranges from 13°C-22°C. As temperatures exceed 13°C, hemp starts an accelerated growth stage (Ehrensing, 1998; Kraenzel et al., 1998). The optimal pH for hemp growth is 6–8.5 in well-drained, loamy soil (Amaducci et al., 2015). Further, soil should be deep, well-aerated, with good water-holding capacity. Four months of frost is required to produce industrial hemp fibers (Ehrensing, 1998). According to the Purdue Industrial Hemp Project, hemp requires calcium concentrations of less than 6,000 ppm, phosphorus concentrations greater than 40 ppm, potassium concentrations greater than 250 ppm, and sulfur concentrations greater than 5,000 ppm (Schumacher et al., 2020).

## Structural Properties of Industrial Hemp

Industrial hemp consists of three types of fibers in its stem: primary bast (outer long fibers), secondary bast fibers, and hemp hurds or shives (inner short fibers). The fibers have very high cellulose content with varying amounts of hemicellulose, lignin, and pectin as its major constituents (Tutt and Olt, 2011). The length of hemp fibers is 1–3 cm, and the bast contains 70.2–74.4% (wt%) of cellulose and only 3.7–5.7% (wt%) lignin (Sisti et al., 2018). By comparison, the length of wood fibers is only 1–3 mm with less cellulose content (40–50%). A single fiber of hemp has a density of 1.48 g/cm^3^ (Sisti et al., 2018) and a tensile strength of 350–800 MPa (Thygesen et al., 2006; Sisti et al., 2018), depending on the climate during the growing season and the genotype, soil type, fertilization, and retting processes. Hemp fibers have an elastic modulus (75 GPa) similar to the stiffness of glass fibers which varies from 50 to 70 GPa (Thygesen et al., 2006). Cellulose and hemicellulose molecules are bound together by lignin molecules. The S2 layer of hemp fiber walls are constructed of 100 mm thick lamellae consisting of one to four cellulose-rich and lignin-poor concentric layers (Thygesen et al., 2006). The inner fibers consist of a secondary xylem fiber network that are shorter (approximately 2 mm) and thinner (6 μm) than the outer fibers and represent approximately 10% of the total fibers (Marrot et al., 2013).

Prior to use as an industrial composite, non-cellulosic materials and lignin are generally removed to expose interfacial hydroxyl groups of cellulose which can be functionalized with other composite materials (Pickering et al., 2007). For example, treatment using alkali conditions removes lignin content by approximately 50% which increases the crystallinity and roughness of the fiber surface. The highest stem biomass is usually yielded if the fiber is harvested prior to the onset of flowering. This phenomenon positively correlates with prolonged durations of the vegetative phase (Salentijn et al., 2019). Hemp fibers harvested early in the blooming stage demonstrate high tensile strength that decreases as the plant matures due to an increase in the proportion of hemps hurds compared to bast (Sisti et al., 2018).

## The Hemp Microbiome

Despite incipient interest in industrial hemp, the microbiome of hemp has yet to be fully characterized. However, emergent, amplicon-based DNA sequencing technologies have made way for the analyses of its microbial constituents. Microbial components (i.e., bacteria, archaea, and fungi) are indispensable factors that help maintain soil and hemp plant health. Microorganisms provide plants with protection from pathogens, nitrogen fixation, and acquisition of micronutrients from the surrounding ecosystem. Plants in turn release life-sustaining nutrients to symbiotic microbes associated with plants. However, due to the decades-long *Cannabis* prohibition, there is a paucity of literature elucidating the composition and function of the hemp microbiome. Hemp plants harbor a robust microbial consortium that are present throughout its life cycle and persists during the retting process (McPartland, 1997; Barnett et al., 2020; Law et al., 2020).

Microbiological retting, or degumming, is the extraction of hemp fibers from harvested stalks that is a traditional and highly widespread retting method. In a recent study, the hemp stalk microbiome was studied during field (dew) retting processes using 16S rRNA next-generation sequencing on the Illumina MiSeq platform. The results demonstrated that the microbiome was largely dominated by endogenous Proteobacteria and showed a proliferation of Bacteroidetes during dew-retting processes (Punja, 2018).

A recent study was conducted using high-throughput sequencing of 16S ribosomal RNA (rRNA) gene (bacteria) and internal transcribed spacer 1 (ITS1; fungal) biomarkers to probe for taxa that comprise the core microbiome of hemp. The study identified operational taxonomic units (OTUs) across four plant compartments/organs (flowers, leaf surface rhizosphere, and root compartment) of *C. sativa* cultivated in six different fields (Barnett et al., 2020). The “core” microbiome was defined as bacterial and fungal OTUs that were ubiquitous in hemp plants and that significantly enriched a plant organ relative to bulk soil. The results demonstrated that hemp did not have a significantly effect on the overall rhizosphere microbial community composition. However, six core bacteria were discovered from root tissue, all of which belong to the phylum Proteobacteria. Eleven core bacteria from the leaf surface were comprised of members of the Proteobacteria, Actinobacteria, and Bacteroidetes phyla. Seven core bacteria from the flower were comprised of members of the Proteobacteria and Firmicutes phyla. All core fungi were classified as Basidiomycota or Ascomycota, or unclassified at the phylum level. Only one core soil rhizosphere fungal OTU, *Bullera albawas*, was identified, but there were 14 core fungal OTUs found on the leaf surfaces and only two core fungi found in the flowers.

In a related study, Wei et al. (2021), also used amplicon-based studies to elucidate the hemp microbiome in 15 plants across 4 hemp ecotypes. The results demonstrated that the Proteobacteria phylum exhibited the highest relative abundance (67.26%) and significant variations in soil and plant compartments. Cyanobacteria and Firmicutes had the greatest abundance in the flower samples, and Proteobacteria, Actinobacteria, and Bacteroidetes were greatly enriched in soil and root samples. Further, *Rhizobium*, *Pseudomonas*, *Bacillus*, and *Sphingomonas* were the top bacterial genera detected in samples, with *Pseudomonas* and *Bacillus* highest in flowers, and *Rhizobium* and *Sphingomonas* highest in stems. Regarding fungi, Ascomycota (50.59%) exhibited the highest relative abundance of fungal taxa.

## Hemp Diseases

Hemp diseases are primarily caused by fungi and rarely by bacteria and viruses (Table 1; McPartland, 1997; Kusari et al., 2013; Giladi et al., 2020; Jerushalmi et al., 2020; Chiginsky et al., 2021; Punja, 2021). Studies have shown that there are 100 true *Cannabis*-related fungal pathogenic species (Punja, 2021). Microbial diseases are present in every growth stage, i.e., from seedling to maturity, and are ubiquitous in every plant organ. Fungal diseases typically cause blight, cankers, leaf spots, mildew, root rot, and wilt. Bacterial diseases generally cause blight, gall, and spots. Viruses and viroid communities cause mosaics and streaks as well as general nutrient deficiency (Chiginsky et al., 2021).

**Table 1:** Common microbial diseases of hemp plants.

Fungi		Bacteria	Viruses
**Anthracnose** *Colletotrichum coccodes* (Wallroth) **Black dot disease** *Epicoccum nigrum* Link **Brown blight** *Acremonium* sp. *Alternaria* spp. (Fr.:Fr.) Keissl. **Brown leaf spot and stem canker** *Ascochyta* spp. *A. prasadii* Shukia and Pathak *Phoma* spp. *Didymella* spp. *P. exigua* Desmaz. *P. glomerata* (Corda) Wollenweb. and Hochapfel *P. herbarum* Westendorp **Charcoal rot** *Macrophomina phaseolina* (Tassi) Goidanich **Cladosporium stem canker** *Cladosporium cladosporioides* (Fresen.) De Vries. *C. herbarum* (Pers.:Fr.) Link *Mycosphaerella tassiana* (De Not.) Johan. **Curvularia leaf spot** *Curvularia cymbopogonis* (C. W. Dodge) roves and Skolko *C. lunata* (Wakker) Boedijn *Cochliobolus lunatus* Nelson and Haasis Cylindrosporium blight *Cylindrosporium* spp. *C. cannabinum* Ibrahimov **Damping-off** *Botrytis cinerea* Pers.:Fr. *Botryotinia fuckeliana* (de Bary) Whetzel *Fusarium oxysporum* Schlechtend.:Fr. *F. solani* (Mart.) Sacc. *Nectria haematococca* Berk. and Broome *Macrophomina phaseolina* (Tassi) Goidanich *Pythium aphanidermatum* (Edson) Fitzp. *P. debaryanum* auct. Non-Hesse *P. ultimum* Trow *Rhizoctonia solani* Kühn *Thanatophorus cucumeris* (A. B. Frank) Donk Downy mildew *Pseudoperonospora cannabina* (Otth) Curzi *P. humuli* (Miyabe and Takah.) G. W. Wils. **Fusarium foot rot and root rot** *F. solani* (Mart.) Sacc. *Fusarium* stem canker *F. sulphureum* Schlechtend. *Gibberella cyanogena* (Desmaz.) Sacc.	**Olive leaf spot** *Cercospora cannabis* K. Hara and Fukui *Pseudocercospora cannabina* (Wakef.) Deighton Ophiobolus stem canker *Ophiobolus cannabinus* Pass. *O. anguillidus* (Cooke in Cooke and Ellis) Sacc. Phoma stem canker *Phoma herbarum* Westendorp. *P. exigua* Desmaz **Phomopsis stem canker** *Phomopsis cannabina* Curzi *P. achilleae* (Sacc.) Hohn. *Diaporthe arctii* (Lasch) Nitschke var. *achilleae* (Auersw.) Wehmeyer Phymatotrichum root rot (Cotton root rot) *Phymatotrichopsis omnivora* (Duggar) Hennebert **Pink rot** *Trichothecium roseum* (Pers.:Fr.) Link **Powdery mildew** *Leveillula taurica* (Lév.) Arnaud. *Oidiopsis taurica* Salmon *Sphaerotheca macularis* (Wallroth:Fr.) Lind *Oidium* sp. **Red boot** *Melanospora cannabis* Behrens (secondary on hemp canker) *Rhizoctonia soreshin* and root rot *R. solani* Kühn **Rust** *Aecidium cannabis* Szembel *Uredo kriegeriana* Syd. and P. Syd. *Uromyces inconspicuus* Otth **Southern blight (Sclerotium root and stem rot)** *Sclerotium rolfsii* Sacc. *Athelia rolfsii* (Curzi) Tu and Kimbrough **Stemphylium leaf and stem spot** *Stemphylium botryosum* Wallroth *Pleospora tarda* E. Simmons *S. cannabinum* (Bachtin and Gutner) Dobrozrakova et al. **Tar spot** *Phyllachora cannabidis* (P. Henn.)	**Bacterial blight** *Pseudomonas syringae* pv. *cannabina* (Sutic and Dowson) Young et al. **Crown gall** *Agrobacterium tumefaciens* (Smith and Townsend) Conn *Striatura ulcerosa* *P. syringae* pv. *mori* (Boyer and Lambert) Young et al. **Xanthomonas leaf spot** *Xanthomonas campestris* pv. *cannabis* Severin	**Alfalfa mosaic and Lucerne mosaic**genus *Alfamovirus, Alfalfa mosaic virus* (AMV) **Arabis mosaic**genus *Nepovirus, Arabis mosaic virus* (ArMV) **Cucumber mosaic**genus *Cucumovirus, Cucumber mosaic virus* (CMV) **Hemp mosaic** *Hemp mosaic virus* *Opuntia-like virus*Hemp streak *Hemp streak virus* *Cryptic virus* **Alfalfa mosaic and Lucerne mosaic**genus *Alfamovirus, Alfalfa mosaic virus* (AMV) **Beet curly top** *Beet curly top virus* **Hemp Vein** *Citrus yellow vein associated virus* **Mitovirus** *Cannabis sativa mitovirus 1 (CasaMV1)* **Dudding** *Hop latent viroid*
**Fusarium wilt** *F. oxysporum* Schlechtend.:Fr. f. sp. *cannabis* Noviello and W. C. Snyder *F. oxysporum* Schlechtend.Fr. f. sp. *vasinfectum* (Atk.) W. C. Snyder and H. N. Hans. **Gray mold** *Botrytis cinerea* Pers.:Fr. **Hemp canker** *Sclerotinia sclerotiorum* (Lib.) de Bary **Leptosphaeria blight** *Leptosphaeria cannabina* Ferraris and Massa *L. woroninii* Docea and Negru *L. acuta* (Fuckel) P. Karst.	**Tropical rot** *Lasiodiplodia theobromae* (Pat.) Griffon and Maubl. **Twig blight** *Dendrophoma marconii* Cav. *Botryosphaeria marconii* V. Charles and Jenkins **Verticillium wilt** *Verticillium albo-atrum* Reinke and Berthier *V. dahliae* Kleb. **White leaf spot** *Phomopsis ganjae* McPartland **Yellow leaf spot** *Septoria cannabis* (Lasch) Sacc. *S. cannabina* Peck		

List of common fungal, bacterial, and viral infections of hemp plants and the etiological taxa that cause the infections (McPartland, 1997; Kusari et al., 2013; Giladi et al., 2020; Jerushalmi et al., 2020; Chiginsky et al., 2021; Punja, 2021).

In 1914, the first published report of microbial infection affecting *C. sativa* was demonstrated in a study by Charles and Jenkins (1914). The fungal disease was evident on fully grown plants and resulted in wilted and drooping foliage that eventually turned brown and died. The pathogen was identified as *Botryosphaeria marconi* (Cav.). It was noted as the first detection of this fungus in America. Since this historical occurrence, two of the most deleterious diseases of outdoor varieties of *C. sativa* have been reported to be infections by phytopathogens *B. cinerea* and *Trichothecium roseum* (McPartland, 1997). A recent Punja (2018), examined flower buds of field-grown *Cannabis* from British Columbia and Alberta in which buds were harvested in 2015–2017. The results of the study demonstrated that internal rot in the bud was associated with a fungal infection caused by *B. cinerea*. Further, *Penicillin olsonii* and *P. copticola* fungi were discovered in pre-harvest flower buds and in dried buds (Punja, 2018). This study also demonstrated *P. olsonii* infection was found in the bracts and stigmas of flower buds.

More recently, a study of Chiginsky et al. (2021) using amplicon-based sequencing to detect the diversity and prevalence of beet curly top as well as virus strains, viral pathogens, and viroid pathogens infecting hemp in Colorado. The phylogenetic results identified cryptic virus, cannabis sativa mitovirus, citrus yellow vein associated virus, opuntia-like virus, and hop latent viroid (Chiginsky et al., 2021). Another recent Punja (2021) publication also listed some of the most important infections that affect indoor cannabis growth. Since fungicides are not currently approved for hemp cultivation, the publication also provides management approaches to combat hemp pathogens. Hemp disease management includes applying anti-fungal and anti-bacterial agents, planting clean stock (mother) plants, adjusting environmental settings to decrease pathogen proliferation, removing diseased cuttings, etc. (Punja, 2021).

## Emerging Topics Involving Industrial Hemp

### Antibacterial Properties of Hemp

An emerging consideration of hemp involves its use to combat the increasing resistance of bacteria to antibiotics. It is a long-held notion that whole hemp seeds inhibit the growth of Gram-positive *Bacillus cereus* and that the resinous flowering tops reduce the growth of other Gram-positive bacilli such as *B. subtilis* as well as *Staphylococcus aureus* (McPartland, 1997). However, the seed does not appear to affect Gram-negative bacteria. A recent study by Blaskovich et al. (2021) confirmed reports that the activity of over 20 types of Gram-positive bacteria, including several strains of the methicillin-resistant *Staphylococcus aureus* (MRSA) pathogens, multidrug-resistant (MDR) *Streptococcus pneumoniae*, *Enterococcus faecalis*, *Clostridioides difficile*, and *Cutibacterium acnes* were inhibited by hemp isolates. Also very interesting, this study demonstrated potent inhibitory activity of a small subset of four Gram-negative bacteria, of particular interest, *Neisseria gonorrhoeae*.

### Pharmaceuticals Derived From Hemp Isolates

Pharmaceutical indications involving oral preparations of CBD oil formulated to effect targets of the central nervous system (CNS) have also emerged as a topic of great interest. Cannabinoids bind to a variety of extracellular targets though cannabinoid G-coupled type 1 receptors (CB1Rs) and type 2 receptors (CB2Rs). CB1Rs are highly expressed in the CNS and effect endocannabinoid system responses such as appetite, dependence, cognition, emotion, memory, motivation, and pain (Piomelli, 2003; Di Marzo, 2008; Basu and Dittel, 2011). CB2Rs are expressed by all cells and play a significant role in potent immune regulation of effector functions, migration in response to endocannabinoids, and proliferation of immune cells (Piomelli, 2003; Di Marzo, 2008; Basu and Dittel, 2011). Prior to the 2018 Farm Bill, it was illegal to grow hemp, and pesticides have not been approved for use. Although there has been significant interest and ubiquitous and often unsubstantiated assertions of the efficacy of products containing CBD, the US Food and Drug Administration (FDA) has approved three foods and only one drug obtained from hemp-derived ingredients contain less than 0.3% THC (Mead, 2019). The approved foods are hulled hemp seed, hemp seed protein powder, and hemp seed oil to be used in human food to treat atypical and severe forms of epilepsy, Lennox–Gastaut syndrome, and Dravet syndrome in patients ages 2 years and older (Mullard, 2018; Mead, 2019; Shen et al., 2021). CBD is prohibited from being marketed as a dietary supplement. Furthermore, foods with added CBD cannot be introduced into interstate commerce according to the FD&C Act. One exception is if the drug product or substance was marketed in foods or dietary supplements prior to its approval and before it was subject to extensive clinical studies (Abernethy, 2019). In 2018, the FDA granted approval of the first and only drug containing CBD-derived substances. The drug, Epidiolex, is an oral solution containing a highly purified liquid formulation of CBD produced by GW Pharmaceuticals in the United Kingdom. A dose of 10 mg/kg of Epidiolex per day must have a purity of no less than 98% (w/w) CBD and less than 0.15% (w/w) THC (Hilderbrand, 2018).

### COVID-19 and Hemp Isolates

In light of the current COVID-19 global pandemic, there has been an emerging demand for new therapies and prevention of the coronavirus infection that has led to millions of mild to fatal disease outcomes worldwide. The SARS-CoV-2 virus is transmitted through respiratory droplets, with possible transmission through aerosol and fomite contact. A recent study reported that CBD inhibits the replication of SARS-CoV-2 through the strong upregulation of genes associated with the host stress response in the early stages of infection in mice (Nguyen et al., 2022). Another recent study suggested that select high-CBD extracts downregulate serine protease *TMPRSS2* gene that produces a crucial transmembrane protein required for SARS-CoV-2 entry into host cells (Wang et al., 2020). The authors suggest that the medical delivery of *Cannabis* should be through capsules, inhalers, or mouth washes, but not through smoking preparations of *C. sativa.* Also, in related studies, tobacco smoking has been shown to exacerbate clinical outcomes of COVID-19. Tobacco smokers compared to non-smokers are 1.4 times more likely to have severe symptoms of COVID-19 and greater than 2.3 times more likely to need mechanical ventilators or die (Guan et al., 2020). Testing needs to be conducted to determine the risk factors associated with different medical modes of delivery of medicinal *Cannabis* for the treatment of COVID-19 (Sexton, 2020).

### Genetic Tools and *C. sativa*

Another topic of rising attention involves the genome editing capabilities facilitated by clustered regularly-interspaced short palindromic repeats (CRISPR/Cas9) technology that holds great promise for targeted improvement of *Cannabis* cultivars. Prior research has yielded hemp plants that contain 0.1% or less THC or single cannabinoids in high concentrations (Small and Marcus, 2003; Wirtshafter, 2004); however, access to the *Cannabis* genome might simplify production of THC-knockout plants *via* CRISPR technology. To date, there are no published accounts of CRISPR-mediated genome editing in *Cannabis*. Nevertheless, there are patents pending for the manipulation of cannabinoid synthesis in hemp using the CRISPR/Cas9 gene editing system to alter cannabinoid-synthesis pathways associated in THC and CBD production (Dolgin, 2019).

## Cannabis in Human Specimens

Since the discovery of major cannabinoids from *C. sativa* L., various forensic analytical tools and protocols have been introduced for the detection, classification, and quantification of naturally occurring cannabinoids (Grijó et al., 2018; Ramirez et al., 2019; Baranauskaite et al., 2020; Moreno et al., 2020; Nuapia et al., 2020; Gerace et al., 2021; Casati et al., 2022). Furthermore, numerous tools and protocols for the extraction cannabinoids from human specimens such as hair, urine, blood, and saliva have also been implemented. As it relates to industrial hemp, analytical methods have primarily been used for forensic differentiation between illegal drug-type *Cannabis* and legal products (i.e., industrial fibers and CBD-rich/THC-poor materials; Hayley et al., 2018).

Forensic qualitative determinations of THC/CBD ratios require unequivocal distinctions between CBD-rich industrial types and THC-rich drug types. Due to strict forensic guidelines, the analytical methods for THC concentration determinations rely on highly specialized and expensive instrumentation. The analytical techniques are based on chromatography methods that separate THC and/or CBD from specimens. The most commonly used techniques are liquid chromatography with tandem mass spectrometry (LC–MS/MS; Ambach et al., 2014; McRae and Melanson, 2020; Karğılı and Aytaç, 2022) and gas chromatography (GC)-MS (Fodor and Molnár-Perl, 2017). Less commonly used separation methods include high pressure liquid chromatography (HPLC) with photodiode array (PDA; Brighenti et al., 2017) and thin layer chromatography (HPTLC)-MS (Corni et al., 2020). Other analytical methods include electrochemical detection of the electroactive groups in CBD using sensors (Cirrincione et al., 2021a).

### Hair

Evidence obtained from hair samples is one of the most important resources in forensic investigations. Hair is an advantageous specimen because it is less invasive compared to collecting urine, blood, and saliva. Additionally, it does not have to be frozen to be stored, and it is harder to adulterate and falsify. Generally, the detection time for *Cannabis* is relatively long for hair samples; it may be detected up to 90 days (Taylor et al., 2017). A solution of hair is commonly screened using Enzyme-Linked Immunoassay Sorbent Assay (ELISA), and the cutoff for a negative THC result is 0.30 pg./mg of hair, depending on the technique.

In contrast to other drugs, hair detection methods have been criticized as having a lack of the sensitivity to function as detectors for cannabinoids. Also, THC present in secondhand cannabis smoke may be incorporated into hair by contamination not consumption. Therefore, new studies have been conducted to create new lines of inquiry for the use of hair to detect THC. A recent forensic study evaluated statistical differences in the CBD/THC ratio in scalp hair samples from 127 individual who reported chronic drug abuse versus samples from industrial hemp growers and seized materials (Casati et al., 2022). Gas chromatography with flame ionization detector (GC-FID) and GC–MS were used to determine metabolic ratios capable of discriminating THC- and CBD-rich cannabis in marijuana consumers’ hair samples. The results determined that CBD/THC ratios can be used as markers able to discriminate between illegal cannabis use and “light” (i.e., low THC/high CBD) use. Another recent study aimed to determine if the consumption of legal (light cannabis) with low THC/high CBD, resulted in positive test results during workplace screening or forensic testing (e.g., roadside or driving relicensing). The study involved the keratin matrix in head and pubic hair roots, and it demonstrated negative results (no accumulation) for the presence of THC in hair samples, but CBD was detected at high concentrations (Gerace et al., 2021).

### Urine

THC is detectable for 3 days to a month or longer in urine (Smith-Kielland et al., 1999; Dolan et al., 2004). The detection depends on how often the person uses marijuana (Dolan et al., 2004). A recent study was performed to determine whether THC, CBD, and cannabinol (CBN) are detected in the urine at 1 and 12 weeks after consuming hemp products (Baeck et al., 2019). GC/MS was used for simultaneous analysis of the three substances and found that consumption does not cause positive *Cannabis* urine test results. Another recent study was conducted to determine whether CBD/hemp products can influence results of urine drug screenings (Spindle et al., 2020). Positive urine drug test results are compared to confirmatory cutoffs in the Mandatory Guidelines for federal workplace drug testing. The results demonstrated that acute administration of 100 mg of oral CBD and 100 mg of vaporized CBD does not generate positive results based on current US drug testing regulations.

### Blood

THC is detectable as soon as 5 h and up to 36 h in blood (Skopp and Pötsch, 2008; Jarvis et al., 2017). Blood testing allows for precise measurements of THC levels and may estimate the dose and the timing of consumption. Blood testing is the preferred method for the interpretation of acute effects after cannabis abuse in emergency situations. Although blood is generally used to detect recent use of THC, there are several apparent disadvantages. The technique is expensive and requires invasive specimen collection by highly trained personnel. Additionally, unlike hair sample collection, venipuncture collection of blood requires suitable venous access and carries a relatively high risk of infection (Hadland and Levy, 2016). In a typical blood specimen, approximately 90% of the THC is distributed in the plasma and the other 10% in red blood cells (Musshoff and Madea, 2006). Furthermore, cannabinoids in blood plasma are approximately twice as concentrated as those in whole blood.

An innovative study evaluated the use of atmospheric pressure chemical ionization for gas chromatography (APGC) coupled to triple quadrupole mass spectrometry (APGC–MS/MS) for the quantitative determination of cannabinoids in human blood serum (Gottardo et al., 2019). The study was conducted to develop and validate new methodologies in accordance with international regulations. The results demonstrated that the technique may separate THC and CBD in less than 10 min. Its limits of quantification were 0.2 ng/ml for THC and 0.4 ng/ml for CBD.

### Saliva

Cannabis is one of the most prevalent drugs detected through saliva testing; it comprises approximately 78% of drug test results (Chatterjee, 2018). THC is detectable for 24 to 48 h in saliva (Dolan et al., 2004; Hadland and Levy, 2016). THC concentrations in saliva may be greater than 1,000 μg/L shortly after smoking cannabis (Lee and Huestis, 2014). A very innovative study by Anizan et al. (2013), compared differences in saliva cannabinoid concentrations from 19 h before to 30 h after smoking a cigarette containing 6.8% THC. The study sampled saliva from frequent and occasional cannabis smokers using the Statsure Saliva Sampler™ Oral Fluid (OD) device. Two-dimensional (2D)-GC–MS was then performed to quantify cannabinoids in saliva specimens. The results showed that saliva specimens were THC-positive for up to 13.5 h after smoking. However, there was no significant difference found between frequent and occasional smokers over 30 h. CBD had up to a 4-h window of detection and between a 6- and 8-h window for CBN.

## Forensic Challenges

The most frequently analyzed forensic samples using CG-MS range from 1.1% detection in samples of human sweat, airborne particles, e-Cigarettes, fingernails, etc. to 28.7% detection in specimens obtained from whole blood, plasma, and serum (Fodor and Molnár-Perl, 2017). There are apparent limitations associated with certain analytical methods. For example, differences in decarboxylation of the non-psychoactive precursor of THC, Δ^9^-tetrahydrocannabinolic acid-A (THC-A), may produce various results when analyzed by HPLC (Dussy et al., 2005). Abiotic factors such as temperature and time resulted in oxidation or ring-opening of THC-A which produced a decrease in total THC yield. Therefore, the authors of the study proposed that the conversion yield should be evaluated with each use of HPLC when analyzed for forensic interests (Dussy et al., 2005).

*Cannabis* chemotypes are largely variable, and drug breeders commonly produce hybrid varieties (Dufresnes et al., 2017). For forensic purposes, the United Nations Office on Drugs and Crimes (UNODC) have provided guidelines for which *Cannabis* plants are classified. The guidelines are based on the concentration of the main phytocannabinoids, specifically THC, CBN, and total CBDs. Using gas chromatographic analysis, if the peak area ratio of [THC + CBN]/[CBD] is less than 1.0, then the cannabis plant is classified as industrial hemp. If the THC/CBD peak area ratio is greater than 1.0, it is classified as marijuana (Tettey et al., 2021). However, in the United States and Canada, as previously stated, only hemp cultivars containing less than 0.3% THC are permitted to be grown for cosmetics (Sun et al., 2021), food (Shen et al., 2021), supplements (Cerino et al., 2021), and textile (Crini et al., 2020). Recent studies have used a high-throughput, spectroscopic-based method using attenuated total reflectance-Fourier’s transform infrared (ATR-FTIR) spectroscopy that has been shown to discriminate fiber-type *Cannabis* from drug-type inflorescences (Cirrincione et al., 2021b).

## Conclusion

It is very timely and important for the scientific community to engage in research to assess the ecological feasibility of hemp once again becoming an American industrial staple. The eco-friendly properties of hemp increase its viability above other less environmentally friendly industrial products. Future studies are expected to elucidate many knowledge and data gaps that currently impede advancing management and production of industrial hemp. For example, in December 2019, the Environmental Protection Agency (EPA) approved the first pesticides for use on industrial hemp (Mark et al., 2020). One of the newly approved pesticides is a conventional insecticide, fungicide, and miticide containing an active ingredient of potassium salts of fatty acids, and nine other biopesticides contain naturally occurring substances that control pests. The approval of these products is the first stride to permit protection for hemp growers.

The Achilles heel of industrial hemp remains its association with marijuana. From the perspective of forensics, a laboratory is required to answer the question, “Does the material presented for analysis contain an illegal THC amount?” The answer to that question must be accurate and reliable using validated methods. Further exacerbating the problem in the USA is the inconsistency of the laws that govern marijuana. It is currently on the controlled substances list, but it is classified for legal use as medical marijuana in 36 states and recreational use in 15 states as well as the District of Columbia (French et al., 2022). Therefore, as the delineation between legally produced hemp and psychoactive forms of *Cannabis* becomes clearer, and with the passage of more regulations to improve growth conditions, research will undoubtedly provide unprecedented opportunities to expand forensic advances and promote development of industrial and therapeutic possibilities for emblematic *Cannabis* products.

## Author Contributions

All authors listed have made a substantial, direct, and intellectual contribution to the work and approved it for publication.

## Funding

While this Review Paper was written, SF, RG, and GJ were funded by the National Science Foundation (NSF) grant HRD 2011764, and GJ was funded by the National Institute of Justice (NIJ) 2017-MU-MU-4042.

## Conflict of Interest

The authors declare that the research was conducted in the absence of any commercial or financial relationships that could be construed as a potential conflict of interest.

## Publisher’s Note

All claims expressed in this article are solely those of the authors and do not necessarily represent those of their affiliated organizations, or those of the publisher, the editors and the reviewers. Any product that may be evaluated in this article, or claim that may be made by its manufacturer, is not guaranteed or endorsed by the publisher.
